# Supplementary material for: Longitudinal Secretion of Paramyxovirus RNA in the Urine of Straw-Coloured Fruit Bats (Eidolon helvum)
Source: Viruses. 2021 Aug 20;13(8):1654. doi: 10.3390/v13081654 (PMC8402643; doi:10.3390/v13081654)
Supplement: Supplementary file 1 [file viruses-13-01654-s001.zip › viruses-1326271-supplementary.pdf]

**Table S1.** Paramyxovirus RNA detections and overlap between the two PCRs. ¶Alone = paramyxovirus RNA was detected using only the one PCR, as indicated. *In one pool, AZ_RMH_9 and AZ_RMH_10A were detected concurrently using the same (RMH) PCR. PAR = PCR using general paramyxovirus primers, RMH = PCR using *Respirovirus-Morbillivirus-Henipavirus* specific primers.

| | **AZ_RMH_2** | **AZ_RMH_9** | **AZ_RMH_10A** | **AZ_RMH_146** | **AZ_RMH_162A** | **AZ_RMH_317A** | **Alone** | **Total** |
|---|---|---|---|---|---|---|---|---|
| AZ_PAR_3 | 1 | 1 | 0 | 0 | 0 | 0 | 3 | 5 |
| AZ_PAR_10B | 0 | 0 | 2 | 1 | 1 | 0 | 11 | 15 |
| AZ_PAR_44 | 0 | 0 | 0 | 0 | 0 | 0 | 2 | 2 |
| AZ_PAR_117 | 3 | 0 | 0 | 0 | 0 | 0 | 0 | 3 |
| AZ_PAR_162B | 0 | 0 | 0 | 0 | 1 | 0 | 1 | 2 |
| AZ_PAR_198 | 0 | 0 | 0 | 0 | 0 | 0 | 2 | 2 |
| AZ_PAR_292 | 0 | 0 | 0 | 0 | 0 | 0 | 2 | 2 |
| AZ_PAR_317B | 0 | 1 | 0 | 0 | 0 | 1 | 1 | 3 |
| Alone¶ | 4 | 7* | 20* | 7 | 3 | 0 | | |
| Total | 8 | 9 | 22 | 8 | 5 | 1 | | |

**Table S2.** Paramyxovirus sequences detected in this study. PAR = PCR using general paramyxovirus primers, RMH = PCR using *Respirovirus-Morbillivirus-Henipavirus* specific primers.

| **Short name** | **Full name** | **GenBank Accession number** | **Accession number and name of the closest relative in the NCBI database** | **% identity with the closest relative** |
|---|---|---|---|---|
| AZ_RMH_2 | BatPV_GH_2019_U2R | MZ393386 | HQ660134.1 Bat Paramyxovirus Eid_hel/GH-M90a/GHA/2009 isolate | 98.86% |
| AZ_RMH_9 | BatPV_GH_2019_U9R | MZ393385 | JN862571.1 Eidolon helvum paramyxovirus clone U54B (L) gene | 97.27% |
| AZ_RMH_10A | BatPV_GH_2019_U10R | MZ393384 | FJ971935.1 Paramyxovirus bat/GH15/2009 (L) gene | 98.40% |
| AZ_RMH_146 | BatPV_GH_2019_U146R | MZ393383 | HQ660147.1 Bat Paramyxovirus Eid_hel/GH-M28/GHA/2009 isolate | 99.77% |
| AZ_RMH_162A | BatPV_GH_2019_U162R | MZ393382 | JN862573.1 Eidolon helvum paramyxovirus clone U51B (L) gene | 99.54% |
| AZ_RMH_317A | BatPV_GH_2019_U317R | MZ393381 | FJ971939.1 Paramyxovirus bat/GH21a/2009 (L) gene | 99.09% |
| AZ_PAR_3 | BatPV_GH_2019_U3P | MZ393377 | JN648061.1 Eidolon helvum paramyxovirus clone U46B (L) gene | 99.25% |
| AZ_PAR_10B | BatPV_GH_2019_U10P | MZ393380 | KF878080.1 Mumps virus strain MuVi/RW154.USA/0.70s[K] | 79.74% |
| AZ_PAR_44 | BatPV_GH_2019_U44P | MZ393379 | JX051320.1 Achimota virus 2 | 99.62% |
| AZ_PAR_117 | BatPV_GH_2019_U117P | MZ393376 | AB853096.1 Bat Paramyxovirus Eid_hel/ZFB09-32/Zambia/2009 L gene | 98.30% |
| AZ_PAR_162B | BatPV_GH_2019_U162P | MZ393375 | JN648082.1 Eidolon helvum paramyxovirus clone U68G (L) gene | 96.80% |
| AZ_PAR_198 | BatPV_GH_2019_U198P | MZ393374 | JX051320.1 Achimota virus 2 | 70.22% |

| | | | | |
|---|---|---|---|---|
| AZ_PAR_292 | BatPV_GH_2019_U292P | MZ393378 | JN648056.1 Eidolon helvum paramyxovirus clone U42A (L) gene | 75.33% |
| AZ_PAR_317B | BatPV_GH_2019_U317P | MZ393373 | HE647836.1 Eidolon paramyxovirus RC09 partial L gene | 97.16% |
